# Developing an interpersonal communication skill scale targeting female nursing students

**DOI:** 10.1186/s13104-020-4896-6

**Published:** 2020-01-28

**Authors:** Junko Kondo, Rie Tomizawa, Tetsuya Jibu, Kei Kamide

**Affiliations:** 1 Tokyo Healthcare University Wakayama Faculty of Nursing, 3 Higashisakanoue-cho, Wakayama, Wakayama 640-8538 Japan; 2 Osaka University Center for Twin Research, 1-7 Yamada-oka, Suita, Osaka 565-0871 Japan; 3 Kansai Welfare Sciences University Department of Healthy welfare, 3-11-1 Asahigaoka, Kashihara, Osaka 582-0026 Japan; 4 Osaka University Graduate School of Medicine, Division of Health Sciences, 1-7 Yamada-oka, Suita, Osaka 565-0871 Japan

**Keywords:** Nursing students’ communication skills, Interpersonal communication, Microcounseling, Communication skills scale, Interpersonal communication assessment scale

## Abstract

**Objectives:**

The purposes of this study are to evaluate the interpersonal communication capabilities in basic nursing education and to develop an interpersonal communication skill scale with reference to microcounseling theory.

**Results:**

A quantitative analytical design was employed that involved administering a 28-item self-efficacy survey with reference to microcounseling techniques to 208s-year female nursing students. Measurement data include the nursing student version of the communication skill preliminary scale draft, the generalized self-efficacy scale, and age. Criterion-related validity was verified through descriptive statistics, exploratory factor analysis, and correlation analysis. Factor analysis resulted in a 4-factor structure based on eigenvalues and scree plot. The reliability coefficient shows a correlation between the total score of each factor and the total score of the generalized self-efficacy scale at the 1% level. On the nursing student communication skill scale, the factor structure comprises four factors and 21 items; adjusting the items by confirming the contents of the question sentences realized the structure of the four factors, which show satisfactory reliability, and 20 scale items. Of these, 18 are classified according to microcounseling techniques. This study demonstrates the content and criterion-related validity of the scale.

## Introduction

The essence of nursing care is focused on interpersonal communication between nurses and patients [1]. Furthermore, the quality of interpersonal communication affects health status, including quality of life and patient satisfaction [2, 3]. Therefore, nurses require the ability to communicate appropriately and effectively with patients, their family members, and work colleagues as a professional [3]. As such, it is necessary for nurses to improve interpersonal communication skills in basic nursing education.

In a previous study on interpersonal communication among nursing students, the components of their capabilities were defined by classifying them into six dimensions (i.e., prescriptive, informative, confronting, cathartic, catalytic, and supportive) [4]. To measure the interpersonal communication capabilities of nursing students, an Interpersonal communication assessment scale (ICAS) was developed based on a literature review and interviews with nursing students [3], that adds Morrison’s six constructs [4] to the following two dimensions: listening and nonverbal skills. This scale evaluates widely interpersonal communication capabilities that nursing students currently possess. Therefore it is only useful for understanding the current status of their skills. However, for nursing education it is necessary to clarify the elements that should be included to improve students’ communication skills in their future education. Thus, the present study focused on the microcounseling theory [5] that includes the eight dimensions shown by the ICAS’ constructs and subdivided capabilities into specific skills. Microcounseling is a theory proposed by Ivey [5]; the various basic techniques common to counselling (i.e., micro-techniques) were extracted, and the relationship between the techniques was summarised in a hierarchical table (Additional file 1: Fig. S1) [6, 7]. Micro-techniques had been used to analyse communication in nursing situations, and corresponds to other communication determined to be beneficial [8]. The efficacy of communication training has been measured using a microcounseling framework [9–11], and an evaluation scale for the specific communication skills created [12].

Given that micro-techniques include content related to attitude, and giving responses in encounters with others, listening techniques to understand the other person [7]; these are skills required for 1st- and 2nd-year nursing students, who often acquire skills for building interpersonal relationships through fundamental clinical practicums on campus. Proactive techniques include feedback, advice, and are necessary for 3rd- and 4th-year nursing students who often experience part of their role as nurses through on-site clinical practice at hospitals. It can be said that these techniques cover the skills necessary for fourth-year nursing education in university. Based on these findings, it appears that the application of this theory was useful for the nursing students’ communication skills.

Thus, the purposes of the present study were to evaluate the interpersonal communication capabilities in basic nursing education and to develop an interpersonal communication skill scale with reference to the theory of microcounseling.

## Main text

### Methods

#### Creation of preliminary scale draft items

A preliminary scale draft was prepared with reference to each unit included in the micro-techniques. Regarding the question items, a total of 28 items were created based on the explanation of each unit [7] (Table 1). The question items consisted of content to confirm a single skill, and when the explanation of each unit included two or more skills, the items were created separately. Because this scale assumes involvement with patients who nursing students meet through opportunities for clinical practice, ‘patient’ was used in place of the term ‘client’ in the content of the question items. The created questions consisted of five units and six items concerning involvement behaviours, six units and eight items concerning listening techniques, and ten units and 14 items concerning proactive techniques.

**Table 1:** The pre-ICSS draft items

No.	Questions	Unit	Techniques
1	Looking at the patient in a way that is natural and not uncomfortable to the patient	Gaze	Involvement behaviours
2	Showing an attitude appropriate for listening (e.g., relaxing the body, sitting straight, and not crossing the arms or legs)	Body language (attitude)	
3	Presenting facial expression appropriate for listening (e.g., calm facial expression)	Body language (facial expression)	
4	Sitting in a way appropriate for listening	Body language (distance)	
5	Nodding while the patient is talking to make it easier for the patient to continue talking	Back channeling (i)	
6	Repeating the last word a patient said to make it easier for the patient to continue talking	Back channeling (ii)	
7	Asking “what kind of” and “why” to encourage the patient’s free response	Opened questions	Listening techniques
8	Asking questions that the patient can answer with “yes” or “no” or with one or two words	Closed questions	
9	Nodding while using back channeling expressions such as “uh-huh” and “I see”	Encouragement (i)	
10	Repeating the key words contained in what the patient has said	Encouragement (ii)	
11	Not simply repeating the words the patient used but rather appropriately expressing what the patient wanted to express using the student’s words	Rephrasing	
12	Focusing on the portions of what the patient is saying related to feelings and repeating the emotion-related words the patient used	Reflecting feelings (i)	
13	Focusing on the portions of what the patient is saying related to feelings and appropriately expressing the patient’s feelings in the student’s words	Reflecting feelings (ii)	
14	Identifying the main point of what the patient said and simply expressing what the patient wanted to convey	Summarizing	
15	Trying to find out how the patient understood his or her problem or how he or she was trying to understand this and repeating this back to the patient as accurately as possible	Reflecting meaning	Proactive techniques
16	Clearly telling the patient what he or she should do	Directions	
17	Telling the patient your ideas to help the patient	Advice	
18	Providing the patient with an explanation of a matter that is easily comprehensible	Explanations	
19	Telling the patient specifically what he or she should do	Instructions	
20	Providing the patient with a view that differs from his or her understanding of the significance of his or her behaviors, ideas, and feelings	Interpretations	
21	Providing the patient with information about the student themselves that is related to the patient in a way that is appropriate to the situation	Self-revelation (i)	
22	When providing information about oneself related to the patient, adjusting the quantity and quality of the information in accordance with the time and place	Self-revelation (ii)	
23	When a patient is about to make a decision, encouraging the patient to think about the good and bad consequences accompanying the decision	Logical consequences	
24	Providing the patient with specific and limited feedback (informing the patient of how he or she appears) while maintaining focus on merits and facts	Feedback (i)	
25	After informing the patient about how he or she appears, confirming whether the information was significant to the patient	Feedback (ii)	
26	Observing the verbal and non-verbal expressions and attitudes displayed by the patient and noticing discrepancies between the two	Confrontation (i)	
27	Appropriately informing the patient of his or her contradictions.	Confrontation (ii)	
28	Checking with the patient to confirm whether the student’s way of handling the patient’s contradictions was effective	Confrontation (iii)	

Furthermore, we inquired into how confident students were in using their skills effectively. This is because they largely consisted of students feeling and evaluating whether communication skills were utilised successfully in the interactions with other persons, and emphasis was placed on confirming the students’ perception of how well they were using the skills rather than the degree of mastery of the skills themselves. In addition, a 4-point Likert scale was adopted. These preliminary scale drafts were examined by two psychology department-affiliated researchers and two nursing department-affiliated researchers; the face validity was ensured through minor adjustments, such as revision of the wording.

#### Examination of criterion-related validity

As previously mentioned, students evaluated whether they have been able to use a large portion of the communication skills effectively in their interactions with their peers. Therefore, we focused on self-efficacy, that is, the belief that one can do something based on past performance [13]. Self-efficacy includes generalized self-efficacy and domain-specific self-efficacy [14]. In the present study, we used the characteristic self-efficacy scale [15], which is the generalized self-efficacy of individuals, to examine the validity of the criteria. This scale is the Japanese version of a self-efficacy scale (SES) [16] and consists of a one-factor structure with 23 items for which reliability and validity have been demonstrated.

#### Study subjects

The subjects comprised 208s-year female students enrolled in three nursing universities in the Kansai region of Japan who were participating in a fundamental nursing clinical practicum.

#### Study design

quantitative analytical study design

#### Measurement data

1. Nursing student version of the interpersonal communication skill preliminary scale draft (i.e., pre-ICSS).
2. SES [15].
3. Age.

#### Data collection method

The survey was conducted from November to December 2014. We visited.

each facility in advance for survey cooperation and conducted surveys at the three nursing universities where research cooperation was possible after the classes were completed. Anonymity was ensured when collecting the questionnaires via collection boxes, which were available for 3 days.

#### Methods of analysis

Descriptive statistics were calculated, exploratory factor analysis (maximum likelihood estimation with promax rotation) was performed, and correlation analysis was conducted to verify criterion-related validity. The suitability of factor analysis was determined by the Kaiser–Meyer–Olkin (KMO) Measure of Sampling Adequacy and Bartlett’s test of sphericity. The mean value of each item was substituted for the missing values. IBM SPSS Statistics version 25 was used for analysis.

### Results

1. Response rate and valid response rate: The response rate was 66.3% (138 responses), and the valid response rate was 96.4% (133 valid responses).
2. Age: The mean age was 20.0 years (standard deviation 0.75), and the range was 19–24.
3. Factor analysis of the pre-ICSS.

#### Determination of factor structure

The KMO measure of sample validity was 0.910 and Bartlett’s test of sphericity was 0.000 (*P* < 0.0001), indicating that the data set was appropriate for factor analysis. Furthermore, because the mean value and distribution of each item were confirmed and neither ceiling effect nor floor effect was noted, we performed analysis on the assumption of a normal distribution. In the factor analysis, items with factor loading of less than 0.4 and items with loading of 0.3 or more across multiple factors were deleted. Factor analysis was performed again with respect to the remaining 21 items, and a 4-factor structure was determined based on eigenvalues and scree plot.

#### Adjustment of items

When confirming the question text for each factor, 18 out of 21 items were classified according to the micro-techniques. Of the three items that fell outside the classification of techniques, the first was No. 9. Regarding this question dividing the two elements of < encouragement > , No. 10 was also created. Because it was synonymous with the No. 5, No. 9 was excluded.

The second was No. 7, which expressed the <opened questions>. This item may be regarded as one of the Involvement behaviours as it encouraged patients to respond freely. In addition, in the hierarchy table in Additional file 1: Fig. S1, [opened and closed questions] was positioned next to [Attending behaviors], and this item was retained because it showed an adjacent relationship as a technique.

The third was No. 15, which expressed the unit <reflecting meaning>. The same factor included a question concerning <reflecting feelings>, which may have been considered a similar response. It was also decided to retain this item.

The above items were arranged based on a discussion involving four researchers (two nursing department and two psychology department-affiliated researchers).

#### Factor analysis for confirmation of factor structure

Factor analysis was performed again with the question sentences, which became 20 items created according to item arrangement and the 4-factor structure was confirmed. These four factors included items such as ‘making facial expressions when trying to listen to the other person’ and ‘nodding while the patient is speaking in order to make it easier for the patient to continue talking’, which were named as involvement techniques; factors including items such as ‘focusing on the emotional part of the patient’s spoken content and appropriately expressing the patient’s emotion in one’s own words’ and ‘repeating important keywords among the words spoken by the patient’ were named reflection techniques; factors including items such as ‘appropriately communicating patient contradictions to the patient’ and ‘providing specific and limited feedback focusing on strengths and facts’ were named proactive techniques (behaviours); and factors including items such as ‘explaining a certain thing so that the patient can understand’ and ‘informing the patient what should be done’ were named proactive techniques (instructions) (Table 2).

**Table 2:** Factor analysis results of the pre-ICSS draft items

No.	I	II	III	IV	Factor name	Cronbachα
						Coefficient
12	1.005	− 0.034	− 0.118	− 0.057	Reflection techniques	0.929
14	0.9	0.034	0.064	− 0.070		
13	0.851	0.1	0.039	− 0.065		
15	0.689	− 0.185	0.221	0.131		
11	0.67	0.137	− 0.018	− 0.111		
16	0.623	− 0.037	0.121	0.244		
3	0.002	0.94	0.052	− 0.196	Involvement techniques	0.867
2	− 0.124	0.811	0.213	− 0.087		
4	− 0.068	0.781	− 0.113	0.197		
1	0.222	0.554	− 0.111	0.132		
5	0.132	0.553	0.031	0.029		
8	0.101	0.49	− 0.103	0.234		
24	0.013	0.018	0.917	0.004	Proactive techniques (behaviours)	0.861
25	0.02	− 0.025	0.836	− 0.029		
23	− 0.011	0.061	0.649	0.072		
28	0.071	0.027	0.558	0.084		
20	− 0.198	− 0.005	0.139	0.889	Proactive techniques (instructions)	0.886
19	− 0.053	0.037	− 0.061	0.889		
18	0.04	0.043	0.059	0.717		
17	0.195	− 0.002	− 0.013	0.661		
	I	II	III	IV		
I	–					
II	0.44	–				
III	0.639	0.31	–			
IV	0.623	0.46	0.6	–		

#### Coefficient of reliability

The alpha coefficient for each factor was 0.884 for the involvement techniques, 0.929 for the reflection techniques, 0.861 for the proactive techniques (behaviours), and 0.886 for the proactive techniques (instructions) (Table 2).

#### Correlation

A correlation between the total score of each factor and the total score of the SES was noted at the 1% level (Table 3).

**Table 3:** Correlation between the pre-ICSS and SES

		SES
		Total
I. Reflection techniques	Pearson’s correlation coefficient	0.254**
	*P*-value (both sides)	0.003
II. Involvement techniques	Pearson’s correlation coefficient	0.398**
	*P*-value (both sides)	0
III. Proactive techniques (behaviours)	Pearson’s correlation coefficient	0.236**
	*P*-value (both sides)	0.006
IV. Proactive techniques (instructions)	Pearson’s correlation coefficient	0.272**
	*P*-value (both sides)	0.002
Pre-ICSS; Total	Pearson’s correlation coefficient	0.373**
	*P*-value (both sides)	0

**P < 0.01

## Discussion

In the pre-ICSS, the factor structure by factor analysis comprised four factors and 21 items; after adjusting the items by confirming the contents of the question sentences, the structure of the four factors and 20 items was realised. The reliability coefficient of the four factors extracted were all 0.800 or higher, which were satisfactory values. In addition, 18 of the 20 items were classified according to the micro-techniques and the remaining two items were also positioned adjacent to each other in the microcounseling hierarchy table or contained synonymous content. In the future, it will be necessary to scrutinise the content, expressions, etc. of these questions to develop the scale, but it appears that content validity was demonstrated by the present study. In addition, a correlation with each of the four factor total scores and the total score of the SES was noted, thereby demonstrating criterion-related validity.

In the present study, the framework of microcounseling including the eight dimensions of interpersonal communication skills indicated by [3] was utilised. Communication skills included interpersonal communication skills had categorised in a multifaceted and systematic manner using ENDCOREs [17, 18]. We aim to clarify the connection between skill classifications created outside the nursing field and the preliminary scales and micro-techniques we have created; in the future, we would like to utilise them as data for examining and evaluating interpersonal communication skills required for nursing students and nurses.

Communication education in basic nursing education is related to the acquisition and improvement of interpersonal communication skills in nursing students. Because communication education requires a theoretical framework [19], it is necessary to examine the evaluation scale of interpersonal communication skills while constructing the framework.

We will continue to create a scale for evaluating and measuring the interpersonal communication skills of nursing students with the aim of connecting it with the evaluation index of educational effects and the creation of an environment for students’ independent learning.

## Limitations

To limit the influencing factors in the creation of the preliminary scale, we analysed only the female nursing students who had no working experience. In nursing schools, there is a significant difference regarding the number of male and female students, and it is therefore difficult to compare gender differences through quantitative research. Furthermore, because the survey was administered to nursing students from three universities, caution is needed in generalising the results.

## Supplementary information

**Additional file 1: Fig S1.** The hierarchical figure of micro-counseling skills (Ivey, 1995). *Excerpts from Fukuhara and Allen EI, Mary BI (2004) Theory and Practice of Micro-counseling.

**Additional file 2**.

## Data Availability

All data generated during this study are included the “Additional file 2” in this published article. The datasets used during the current study are available from the corresponding author on reasonable request.
