# Supplementary material for: Developing an interpersonal communication skill scale targeting female nursing students
Source: BMC Res Notes. 2020 Jan 28;13:43. doi: 10.1186/s13104-020-4896-6 (PMC6988265; doi:10.1186/s13104-020-4896-6)
Supplement: Supplementary file 1 — Additional file 1: Fig S1. The hierarchical figure of micro-counseling skills (Ivey, 1995). *Excerpts from Fukuhara and Allen EI, Mary BI (2004) Theory and Practice of Micro-counseling. [file 13104_2020_4896_MOESM1_ESM.docx]

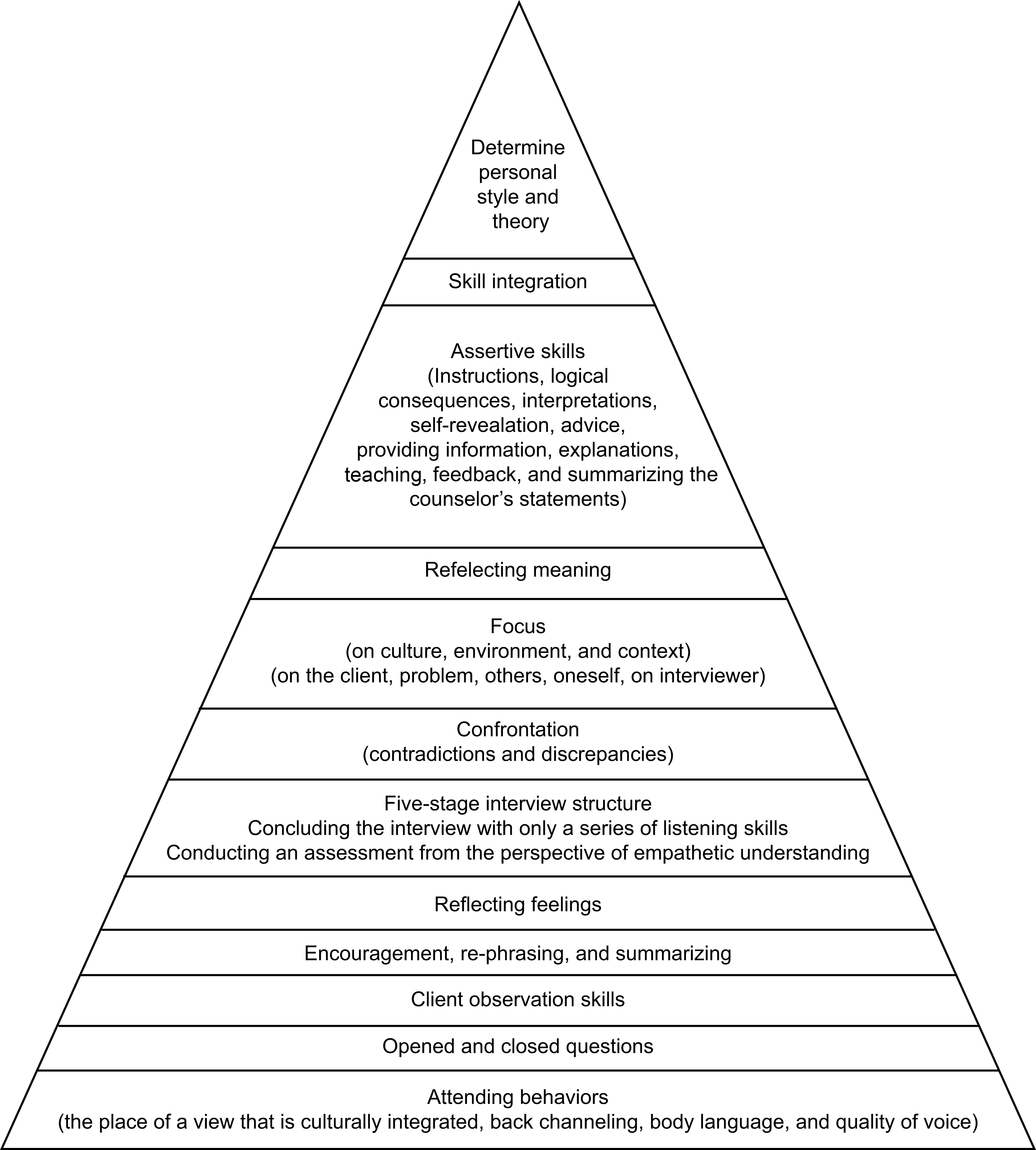


**Fig S1:** The hierarchical figure of micro-counseling skills (Ivey, 1995)

*Excerpts from Fukuhara and Allen EI, Mary BI (2004) Theory and Practice of Micro-

counseling.
